# The Peritoneum Is Both a Source and Target of TGF-β in Women with Endometriosis

**DOI:** 10.1371/journal.pone.0106773

**Published:** 2014-09-10

**Authors:** Vicky J. Young, Jeremy K. Brown, Philippa T. K. Saunders, W. Colin Duncan, Andrew W. Horne

**Affiliations:** MRC Centre for Reproductive Health, The University of Edinburgh, Queen’s Medical Research Institute, Edinburgh, United Kingdom; Northwestern University, United States of America

## Abstract

Transforming growth factor-β (TGF-β) is believed to play a major role in the aetiology of peritoneal endometriosis. We aimed to determine if the peritoneum is a source of TGF-β and if peritoneal TGF-β expression, reception or target genes are altered in women with endometriosis. Peritoneal fluid, peritoneal bushings and peritoneal biopsies were collected from women with and without endometriosis. TGF-β1, 2 and 3 protein concentrations were measured in the peritoneal fluid. TGF-β1 was measured in mesothelial cell conditioned media. Control peritoneum and peritoneum prone to endometriosis (within Pouch of Douglas) from women without disease (n = 16) and peritoneum distal and adjacent to endometriosis lesions in women with endometriosis (n = 15) and were analysed for TGF-β expression, reception and signalling by immunohistochemistry, qRT-PCR and a TGF-β signalling PCR array. TGF-β1 was increased in the peritoneal fluid of women with endometriosis compared to those without disease (*P*<0.05) and peritoneal mesothelial cells secrete TGF-β1 in-vitro. In women with endometriosis, peritoneum from sites adjacent to endometriosis lesions expressed higher levels of *TGFB1* mRNA when compared to distal sites (*P*<0.05). The TGF-β-stimulated Smad 2/3 signalling pathway was active in the peritoneum and there were significant increases (*P*<0.05) in expression of genes associated with tumorigenesis (*MAPK8, CDC6*), epithelial-mesenchymal transition (*NOTCH1*), angiogenesis (*ID1, ID3*) and neurogenesis (*CREB1*) in the peritoneum of women with endometriosis. In conclusion, the peritoneum, and in particular, the peritoneal mesothelium, is a source of TGF-β1 and this is enhanced around endometriosis lesions. The expression of TGF-β-regulated genes is altered in the peritoneum of women with endometriosis and this may promote an environment favorable to lesion formation.

## Introduction

Endometriosis is a non-malignant disorder defined by the presence of endometrial tissue outside the uterus, associated with debilitating pelvic pain and impaired fertility [1]. The disease is estimated to affect 10–15% of women of reproductive age, with an annual cost recently estimated at $12722, per women [2]. Endometriosis is currently managed surgically or medically but symptoms can recur in up to 75% of surgical cases and available medical treatments have undesirable side effects [3], [4]. A major obstacle in developing new treatment strategies for endometriosis is our limited understanding of the aetiology of this disease.

Although shed endometrium and retrograde menses are widely believed to play a role in the aetiology of this enigmatic disorder there is also increasing evidence that the mesothelial cells lining the peritoneal cavity may also contribute to the development and maintenance of peritoneal endometriosis [5]. Studies have shown that mesothelial cells provide a surface for ectopic endometrial cell to attach [6]–[8], and it has been reported that they facilitate ectopic endometrial cell invasion through tissue remodelling [9], [10] and/or through changes in mesothelial cell morphology [11], [12]. Peritoneal mesothelial cells secret a wide-range of pro-inflammatory cytokines and growth factors that may promote increased cell proliferation and angiogenesis [10], [13]. Importantly they may also have impacts on immune cell function, allowing ectopic endometrial cells to evade scavenger cells [14]. Notably, a study by Hull et al. showed that peritoneal mesothelial cells, together with other host derived cells, were incorporated into peritoneal-like lesions in a mouse model of endometriosis demonstrating that they may contribute to the cellular constitutes of endometriosis lesions [15].

Transforming growth factors β (TGF-β) is a multifunctional growth factor that is responsible for regulating cell proliferation, differentiation, angiogenesis and immune responses. In the context of the current study these TGF-β mediated processes are all considered to play a role in endometriosis lesion formation [16]. There are three TGF-β ligands (TGF-β1, TGF-β2, TGF-β3), which have been shown to have overlapping functions. The ligands are secreted as latent precursor molecules and activated by proteolytic cleavage. Once activated the TGF-βs exert their effects through binding and coupling of TGF-β receptors I and II, which in turn phosphorylate the intracellular factors Smad2 and/or 3 that regulate transcription of target genes [17], [18].

Dysregulated TGF-β signalling has been implicated in several pathologies and there is growing evidence that TGF-β may play a role in the aetiology of endometriosis. Several studies have reported women with endometriosis to have increased levels of TGF-β in their peritoneal fluid and serum, when compared to women without disease and levels appeared to change across the menstrual cycle [19]–[21]. More recently, TGF-β1, and its signalling targets involved in cell survival, were reported to be altered in the eutopic endometrium of women with endometriosis when compared to women without disease [22]. Decreased NK cell activity within the peritoneal cavity in women with endometriosis has been attributed to increasing concentrations of TGF-β [23]. Interestingly, TGF-β1 was identified as central component of one of four molecular endometrial/peritoneal networks identified in a model of endometriosis and *TGFB1* transcripts from both the host (mouse) and endometrial (human) compartments appeared to contribute to lesion development [24]. In a follow up study using a mouse model of endometriosis, Hull et al showed *TGFB1*-null mice, developed fewer and smaller peritoneal endometriosis-like lesions than their wild-type counterparts [15]. However, the peritoneum as source of TGF-β has yet to be defined in the context of endometriosis. Furthermore the impacts of increasing TGF-β concentrations on the development and progression of endometriosis remains poorly understood.

In this study, we aimed to determine if the peritoneum is a source of TGF-β and if peritoneal TGF-β expression and/or reception are altered in women with endometriosis. We also aimed to investigate the expression profile of key TGF-β target genes in women with and without endometriosis.

## Materials and Methods

### Subjects

All of the tissues used in this study were collected with informed written consent from women undergoing laparoscopic investigation for chronic pelvic pain, to identify underlying endometriosis under ethical approval obtained from the Lothian Research Ethics Committee (LREC 11/AL/0376). All of the women included in the study were aged between 18–45 years, had regular 21–35 day menstrual cycles and none of them were taking hormonal treatments. The ‘control’ group of women had no evidence of endometriosis at laparoscopy, nor was there evidence of any other underlying pelvic pathology to explain their painful symptoms (e.g. adhesions). The women with endometriosis had macroscopic evidence of disease at laparoscopy and this was later confirmed by histology.

Peritoneal fluid, primary peritoneal mesothelial cells (HPMCs) and peritoneal biopsies were collected at the start of surgery from women with and without endometriosis. Peritoneal fluid (5–10 ml) was collected from women with (n = 6) and without (n = 6) endometriosis and stored in cryovials at −80°C for later analysis. HPMCs (n = 3) were isolated at the time of surgery as previously described by gentle brushing the pelvic mesothelium with a Tao™ brush followed by vigorously agitating in 15 ml of serum-containing culture media to dislodge cells before transferring to a 75 cm^2^ culture flask and incubated at 37°C under 5% CO_2_ in air (QC Sciences, Virginia, USA) [25].

In women without endometriosis we biopsied peritoneal tissue (0.5 cm diameter) from sites ‘prone’ to developing endometriosis lesions, within the Pouch of Douglas (n = 8), and control locations taken 2–3 cm outwith the pelvic brim (n = 13), prone sites were determined in accordance with a study by Mahmood and Templeton 1991 [26]. In women with endometriosis, we biopsied peritoneal tissue from sites adjacent to endometriosis lesions, taken 2–3 cm from lesions (n = 3), and at control sites distal to endometriosis lesions, taken 2–3 cm outwith the pelvic brim and in the same position as the control biopsies from women without disease (n = 11). In women with and without endometriosis we collected serum and endometrial biopsies for confirm cycle stage. The tissues were collected according to the Endometriosis Phenome and Biobanking Harmonisation Project (EPHect) guidelines (http://endometriosisfoundation.org/ephect/).

Upon collection, peritoneal biopsies were divided into two with half immersed in RNAlater at 4°C for 24 hrs before storage at −80°C prior to RNA extraction, and half fixed in 4% neutral-buffered formalin (NBF) for 24 hrs at 4°C before storing in 70% ethanol prior to embedding in paraffin wax for histological studies. All peritoneal biopsies collected were studied histologically to confirm the absence of endometriosis tissue.

### Cycle staging

Cycle phase was confirmed: (1) by date of last menstrual period, (2) by the measurement of serum estradiol and progesterone levels, and (3) by examination of endometrial biopsies (stained with haematoxylin and eosin) collected at the same time as the peritoneal fluid samples. The samples were examined by an expert histopathologist who used Noyes’ criteria to determine cycle phase. All samples included in this study were in the luteal phase of the cycle.

### Establishment of primary human peritoneal mesothelial cell (HPMC) cultures and experimental treatment of HPMC

Brushings of HPMC were cultured in HOSE1 media containing; 40% media 199, 40% MCDB 105 and supplemented with 15% FBS, 0.5% penicillin/streptomycin and 1% L-glutamine, as previously described [25] (Life Technologies Inc., Paisley UK and Sigma Chemical Co., Poole UK). For immunoassay, cells were cultured in serum free conditions for between 12 and 48 hrs. HPMC conditioned culture media was then removed and stored at −80°C for later analysis by immunoassay. Three technical replicates were performed for each patient sample and a total of 3 patient samples were included in this analysis.

### TGF-β1, TGF-β2 and TGF-β3 Immunoassay

TGF-β1 ELISA was performed using the Human TGF-β1 Quantikine kit (DB100B), and TGF-β2 and TGF-β3 ELISAs were performed using the Human TGF-β2 (DY302) or Human TGF-β3 (DY243) ELISA Duo set, all according to manufacturers instructions (R&D systems, Abingdon UK). Samples were assayed for active and total TGF-β1-3 levels. For total levels, sampled were pre-treated with 1 M HCL for 10 mins before neutralizing with 1.2 M NaHO/0.5 M HEPES buffer. ELISA plates were read using Lab Systems Multiscan EX Microplate reader at 450 nm with wavelength correction at 540 nm. Samples were quantified using standard curve analysis within the linear range of 2000 pg/ml to 16 pg/ml. Intra-assay CV was 2.5% and the between batch CV was 8.3% for cell culture supernatants.

### Immunostaining

Five micron sections of paraffin embedded tissue were mounted onto electrostatically charged microscope slides and dewaxed in xylene (2×5min) and rehydrated (VWR, Leicestershire, UK). Antigens were retrieval by pressure-cooking slides in 10 mM Tris 1 mM EDTA pH 9, for 5 mins. Slides were washed in H_2_O before incubation with 3% hydrogen peroxide for 30 min (Sigma). Slides were blocked in either normal horse serum diluted 1∶12 in Tris buffered saline with 0.5% Tween 20 (TBST20) or in 5% BSA in Tris buffered saline with 0.5% Tween 20; for 30 mins. Incubation with primary antibody diluted in blocking buffer was performed overnight at 4°C with either; rabbit anti-TGF-β1 (Abcam Ab9758 1∶1000), rabbit anti-TGF-βR1 (Santa Cruz 398 1∶200), rabbit anti-TGFβR2 (Santa Cruz 220 1∶2000), rabbit anti-pSmad2/3 (Santa Cruz 10790 1∶1000) or equimolar concentrations of rabbit IgG (Dako X0903) as a negative control (Santa Cruz, Heidelberg, Germany, Abcam, Cambridge UK; Dako, Cambridge, UK and Biosera, Uckfield, UK). Slides were washed in TBST20 and incubated for 30 min with species specific impress kit and washed again before incubation with 3, 3′-diaminobenzidine for 5 min (Vector Laboratories). Slides were then counterstained with hematoxylin, dehydrated and visualized by light microscopy, using an Olympus Provis microscope equipped with a Kodak DCS330 camera (Olympus Optical Co., London, UK, and Kodak Ltd., Herts, UK). A total of 8 peritoneal biopsies (n = 2 each group) were examined using immunohistochemistry in total. Due to the limited supply of peritoneal tissue, both positive and negative controls were performed on menstrual stage endometrial tissue.

### mRNA transcript analysis

All peritoneal biopsies assayed for mRNA transcript analysis were from the luteal phase of the cycle. RNA was extracted from peritoneal biopsy tissue using the RNeasy Mini Kit with on-column DNase1 digestion (Qiagen, Manchester UK). First-strand cDNA synthesis was performed using Superscript VILO Master Mix (Life Technologies Inc.). Both processes were performed according to the manufacturer’s instructions.

Quantitative (q)RT-PCR was performed with brilliant III ultra-fast SYBR green qRT-PCR master mix using an ABI Prism 7900 Fast system under standard running conditions with pre-validated primers shown in Table 1 (Applied Biosystems, Warrington, UK, Agilent, Berkshire, UK and Primerdesign). Messenger RNA transcripts for TGF-β1 (*TGFB1*), TGF-βR1-2 (*TGFBR1 TGFBR2*) and Smad 3 (*SMAD3*) were quantified using the 2^−ΔCt^ method relative to GAPDH, as determined by geNorm housekeeping assay (Primerdesign).

**Table 1:** Primers used for qRT-PCR including primer sequence.

Gene of interest	Direction	Primer sequence
*TGFB1*	Sense	CACTCCCACTCCCTCTCTC
	Anti sense	GTCCCCTGTGCCTTGATG
*TGFBR1*	Sense	TGACTGAAGGCTGCTCTGG
	Anti sense	CATCTGCTCAATCTCCAAACTTG
*TGFBR2*	Sense	TCCTTCAAGCAGACCGATGT
	Anti sense	GAACCAAATGGAGGCTCATAATC
*SMAD3*	Sense	GGCTGCTCTCCAATGTCAAC
	Anti sense	ACCTCCCCTCCGATGTAGTA

All primers were pre-validated and supplied by Primer Design.

### TGF-β signalling PCR array

The PCR array plate included 84 TGF-βregulated genes involved in functional processes such as differentiation, proliferation, migration, apoptosis and cell cycle control. All peritoneal biopsies assayed in the TGF-β signalling PCR array were matched samples from the luteal phase of the cycle. RNA was extracted from peritoneal biopsy samples from women without endometriosis at control sites (n = 3) and sites prone to endometriosis (n = 3) and from women with endometriosis at sites distal (n = 3) and adjacent (n = 3) to endometriosis lesions. RNA was adjusted to 125 ng/ml and reverse transcribed using the RT^2^ single strand cDNA synthesis kit according to manufacturers instructions (Qiagen). RT-PCR reactions were prepared using RT^2^ qPCR master mix using an ABI Prism 7900 Fast system according to the manufacturer’s instructions. Results were quantified using the web portal:

http://www.SABiosciences.com/pcrarayanalysis.php.

### Statistical analysis

All the results are expressed as mean ± standard error of the mean of a minimum of 3 independent experiments. ELISA data was analyzed using unpaired students *t* test after testing for normal distribution. Quantitative RT-PCR was analyzed using paired and unpaired students *t* tests, as appropriate after testing for normal distribution. All statistical results were generated using GraphPad PRISM version 5 statistical software (GraphPad Software Inc, San Diego, USA) and a *P* value of <0.05 was considered significant.

## Results

### TGF-β1 concentrations are increased in the peritoneal fluid of women with endometriosis and the peritoneal mesothelium synthesizes and secretes TGF-β1

**Figure 1:**
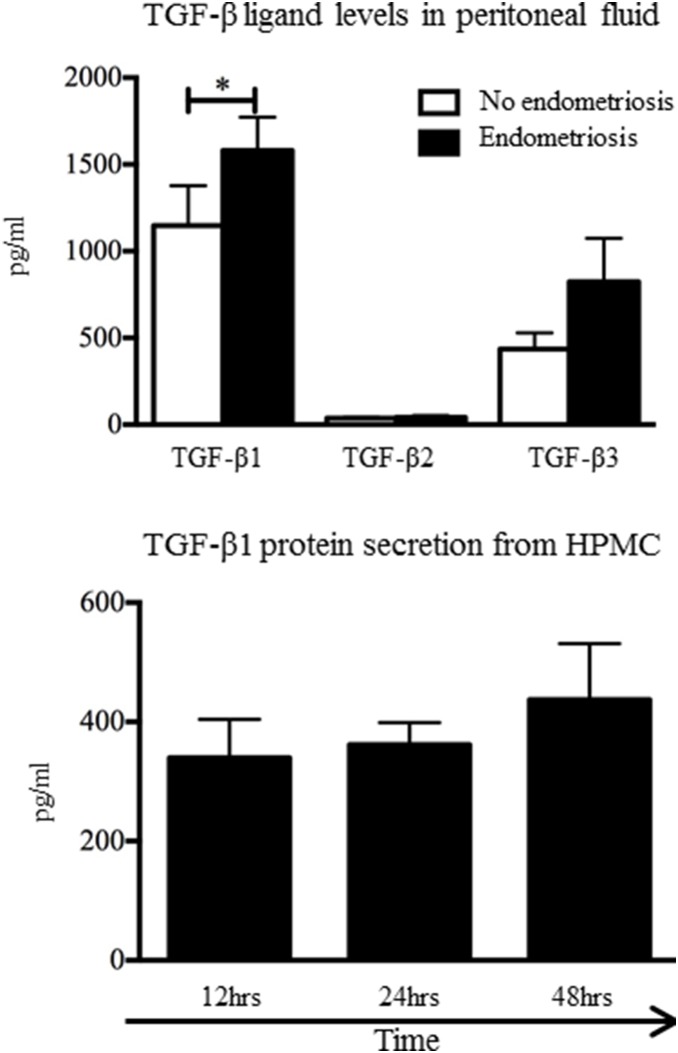
All TGF-β ligands are expressed in peritoneal fluid from women with and without endometriosis. TGF-β1 was significantly increased in peritoneal fluid from women with endometriosis compared to women without. Levels of TGF-β2 do not change between women with and without endometriosis. TGF-β3 levels appear to increase in women with endometriosis however this is not a significant change. Cultured HPMCs secreted TGF-β1 protein in-vitro. n = 12 peritoneal fluid, n = 3 HMPC (*p<0.05).

We assayed active and total concentrations of TGF-β1, TGF-β2 and TGF-β3 in the peritoneal fluid of women with and without endometriosis in the luteal phase of the menstrual cycle. Active concentrations of TGF-β1-3 were undetectable in peritoneal fluid; therefore we used total concentrations only in this analysis. We found all TGF-β ligands to be present in the peritoneal fluid of women with and without endometriosis (Figure 1A). TGF-β1 was significantly increased in the peritoneal fluid of women with endometriosis when compared to women without (Figure 1A). We found no significant changes in TGF-β2 or TGF-β3 levels between women with and without endometriosis (Figure 1A). To determine if the peritoneum is source of TGF-β1, we measured TGF-β1 protein in conditioned media from primary cultures of HPMCs (Figure 1B). We found HPMCs to secret TGF-β1 protein and this appeared to increase over time, although no significant difference was found with the time points analysed (Figure 1B).

### TGF-β1 and its receptors together with phosphorylated Smad 2/3 are localized to the mesothelial cells

**Figure 2:**
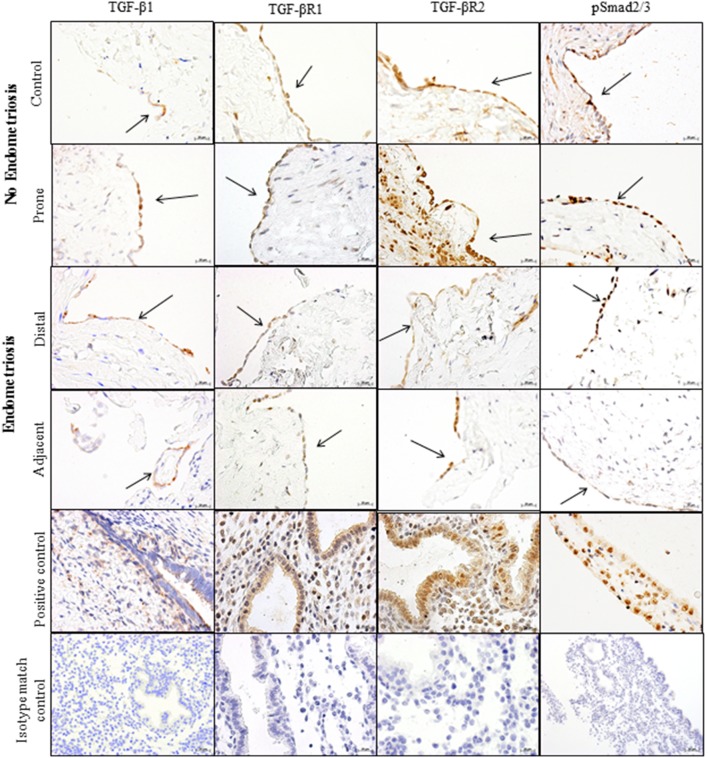
TGF-β1, TGF-βR1, TGF-βR2, and pSmad 2/3, are localized to the mesothelial cells of the peritoneum of women without endometriosis at contol sites and prone sites. TGF-β1, TGF-βR1, TGF-βR2, and pSmad 2/3, are localized to the mesothelial cells of the peritoneum of with endometriosis at sites disteal and ajacent to endometriosis lesions. No staining was observed in the negative control sections. *n = 8.*

We next examined peritoneal biopsies for presence and localization of TGF-β1 by immunohistochemistry. TGF-β1 was present in peritoneum from women with and without endometriosis and was localized to peritoneal mesothelial cells (Figure 2). To investigate whether the peritoneum is receptive to TGF-β signalling, peritoneal biopsies from women with and without endometriosis were examined for presence and localization of TGF-β receptors 1 and 2 and phosphorylated Smad 2/3 (pSmad 2/3) by immunohistochemistry. TGF-β receptors 1 and 2 and pSmad 2/3 (Figure 2) were localized to the peritoneal mesothelial cells of peritoneal biopsies from women without disease (Figure 2) and women with disease (Figure 2). There was no staining observed in the isotype matched control sections (Figure 2).

### The peritoneum adjacent to endometriosis lesions expressed increased TGFB1

**Figure 3:**
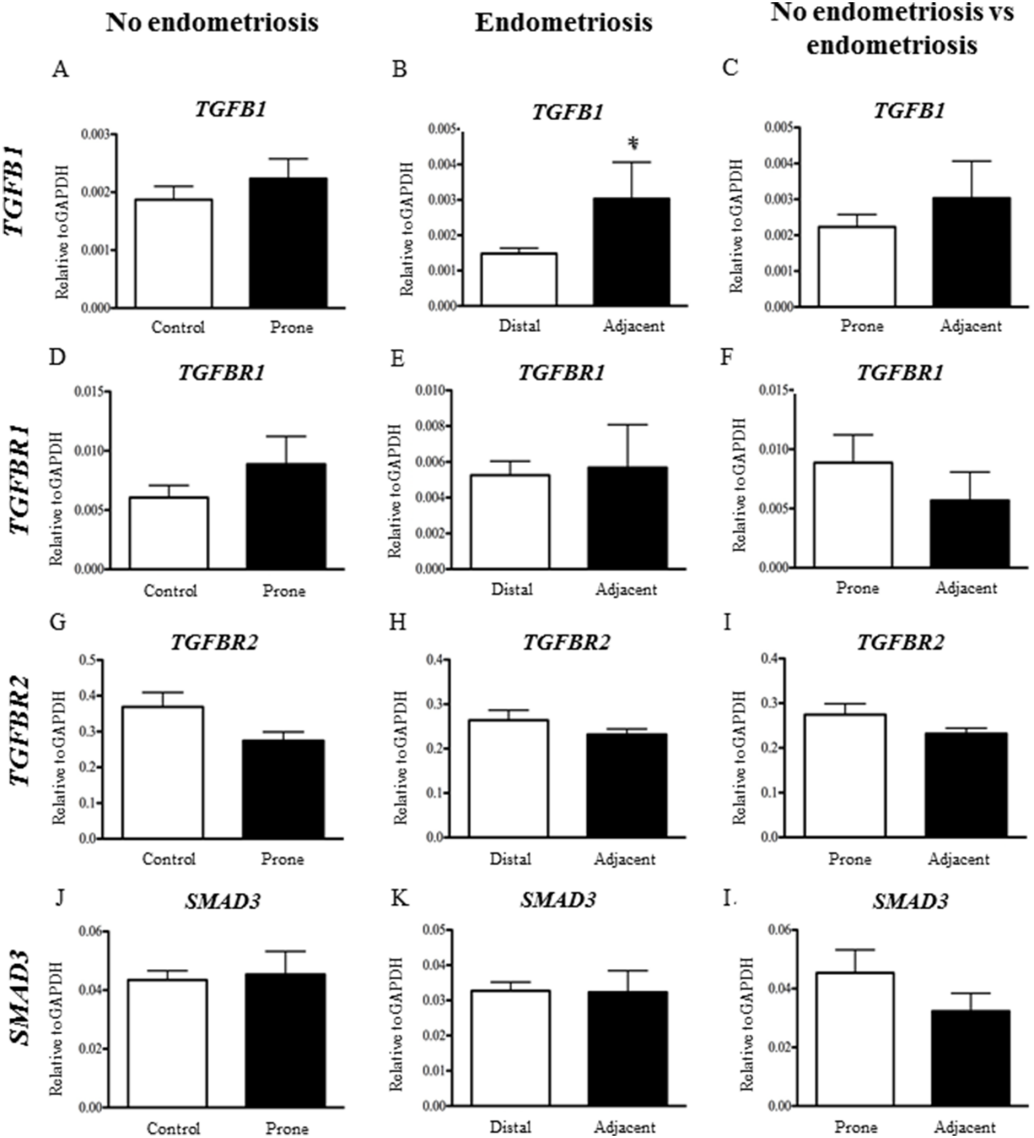
*TGFB1* mRNA expression was significantly increased in the peritoneum adjacent to endometriosis lesions compared to peritoneum distal to lesions in women with endometriosis (B). There was no signifianct difference in TGFB1 expression between women with and without endometriosis, nor was there a difference in expression between control and prone sites of peritoneum in women without endometriosis. There is no significant change in mRNA expression for *TGFBR1, TGFBR2* and *SMAD3* in all comparisons made (A, C–L). n = 35 (*p<0.05).

To investigate if the peritoneal source of TGF-β1 or its signalling components, TGF-βR1, TGF-βR2 and Smad3, are altered in women with endometriosis, we quantified mRNAs encoded by *TGFB1, TGFBR1, TGFBR2* and *SMAD3*. Notably *TGFB1* mRNAs were significantly increased (p<0.05) in peritoneum adjacent to endometriosis lesions when compared to peritoneum that was distal to the lesion (Figure 3B), but there was no change in TGFB1 expression between women with endometriosis and those without (Figure 3C) or between sites of peritoneum in women without disease (Figure 3A). There was no significant changes in expression of *TGFBR1, TGFBR2* or the downstream regulator *SMAD3* in all comparisons made (Figure 3 A, C–L), suggesting that responsiveness to TGF-β ligands is maintained adjacent to the lesion.

### The expression of several TGF-β-regulated genes is increased in peritoneum adjacent to endometriosis lesions

We investigated the expression of TGF-β signalling targets in peritoneal biopsies from women with and without endometriosis in the luteal phase of the menstrual cycle, using a commercial TGF-β target PCR array. We made three comparisons using this methodology. Firstly, we compared peritoneum from sites prone (n = 3), to control sites (n = 3), to developing endometriosis, in women without endometriosis. As endometriosis is most commonly found within the pelvic brim, we made this comparison to determine if there was any difference in the peritoneal tissue at this location that may predispose to the development of endometriosis [26].

Secondly, we compared peritoneum from women with endometriosis from sites adjacent (n = 3) and distal (n = 3) to endometriosis lesions. We made this comparison to determine if there was any difference in the peritoneal tissue at this location that may contribute to the development of endometriosis.

Finally, we compared peritoneum from women with endometriosis at sites adjacent to endometriosis lesions (n = 3) to peritoneum from women without disease at sites prone to endometriosis (n = 3). We made this comparison to determine if there was any difference in the peritoneal tissue between women with and without endometriosis.

**Figure 4:**
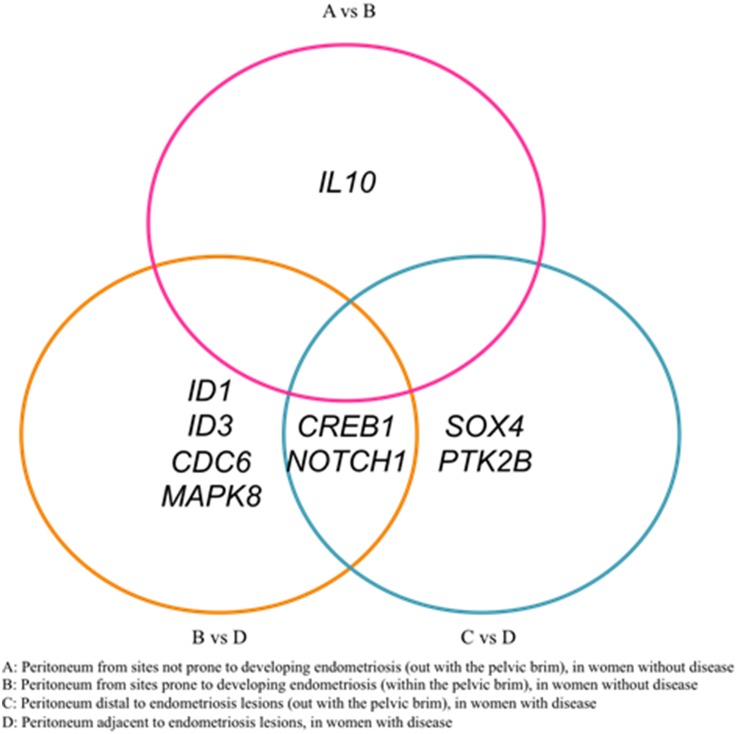
Venn diagram displaying significant changes in gene expression of TGF-β signalling target in the 3 comparisons made between women with and without endometriosis.

The overall expression profile for all array candidate genes can be found in Data S1. Of the 82 genes analysis, we found 3 genes (*AIPL1, MYOD1, SHH*), to be undetectable in all samples. Only one gene, *IL10*, was significantly increased at the sites prone to endometriosis when compared control peritoneal (fold change 2.75, p = 0.026) (Figure 4).

When we compared peritoneum from women with endometriosis to peritoneum from women without we found six genes to have significant changes in expression (Table 2, Figure 4). These genes included *NOTCH1* involved in cell development and TGF-β induced EMT [27] and *CREB1* associated with neurogenes [28]. *ID1* and *ID3*, associated with cell differentiation, angiogenesis and reported to be increased in several cancers [29]; *MAPK8* associated with cell cycle control and cell differentiation [30] and *CDC6* a protein involved in DNA replication and which has been implicated in EMT and cancer development [31].

**Table 2:** Table displays gene expression including fold change and p value of TGF-β signalling target genes in peritoneal biopsies from women with endometriosis compared to women without at sites prone and adjacent to endometriosis lesions.

Gene	Symbol	Fold Change	p Value
CAMP responsive elementbinding protein 1	*CREB1*	1.27	0.009
Notch 1	*NOTCH1*	2.46	0.011
Mitogen-activated proteinkinase 8	*MAPK8*	1.31	0.037
Inhibitor of DNA bindingprotein 1	*ID1*	2.73	0.039
Inhibitor of DNA bindingprotein 3	*ID3*	1.72	0.041
Cell division cycle 6 homolog	*CDC6*	3.18	0.046

*n = 6.*

Finally we found four genes to be significantly increased in the peritoneum adjacent to endometriosis lesions in women with endometriosis compared to peritoneum distal to the lesion (Table 3, Figure 4). These were *NOTCH1* and *CREB1* as previously mentioned; *SOX4* a transcription factor that is increased during EMT and heavily associated with tumorigenesis and metastasis [32] and *PTK2B* essential in neuropeptide signalling [28].

**Table 3:** Table displays gene expression including fold change and p value of TGF-β signalling target genes in peritoneal biopsies from women with endometriosis at sites ajacent compared to sites distal to endometriosis lesions.

Gene	Symbol	Fold Change	p Value
CAMP responsive elementbinding protein 1	*CREB1*	1.44	0.026
Notch 1	*NOTCH1*	1.37	0.024
Sex determining region Y-box 4	*SOX4*	1.76	0.004
PTK2B protein tyrosine kinase 2 beta	*PTK2B*	−1.46	0.003

*n = 6.*

## Discussion

In this study, we have demonstrated that TGF-β1 is increased in the peritoneal fluid of women with endometriosis, and that TGF-β1 and TGF-β signalling targets associated with cell cycle control, angiogenesis, EMT and tumorigenesis are significantly increased in the peritoneum of women with endometriosis compared to those without. We believe that these changes may help establish an environment favourable for endometriosis lesion formation within the peritoneum contributing to the aetiology of this disease.

Peritoneal fluid levels of TGF-β1 is significantly increased in women with endometriosis during the luteal phase. These results are in agreement with several studies reporting TGF-β and TGF-β1 levels to be increased in women with endometriosis and also to be increased across the menstrual cycle [19]–[21]. In only one pervious study [33] did the authors indicate if total or bioactive levels of TGF-β were measured, another did not differentiate between the TGF-β ligands assayed [34]. In the current study we have extended these findings reporting bioactive TGF-β1, 2 and 3 are undetectable in the peritoneal fluid, suggesting that they may only have an effect following activation within the peritoneum or lesions. A previous study examined activation of TGF-β in women with endometriosis via the plasminogen activation pathway that was found to be increased at sites of endometriosis lesions [35].

In this study, we demonstrated that the HPMC secreted TGF-β1 protein and these cells may contribute to the significant rise in TGF-β1 in the peritoneal fluid of women with endometriosis. Peritoneal mesothelial cells are known to overexpress TGF-β1 ligands into the peritoneal fluid in response to peritoneal inflammation [36] and this has been linked to pathologies such as peritoneal fibrosis and cancer. Furthermore, this increase in TGF-β1 ligand expression may have direct effects on adjacent endometriotic cellular activity. For example, TGF-β1-null mice had significantly reduced numbers of macrophages within endometriosis-like lesions, when compared to wild-type mice, suggesting that TGF-β1 plays a critical role in macrophage recruitment to lesions [15]. Furthermore, decreased natural killer cell activity within the peritoneal cavity in women with endometriosis has been attributed to increasing concentrations of TGF-β within the peritoneal fluid [23]. To date, it is not yet known if the increased levels of peritoneal fluid TGF-β1 precedes or follows the development of endometriosis. However, as retrograde menstruation and the presence of endometrial cells within the peritoneal cavity can induce inflammation, the development of endometriosis and the increase in TGF-β1 are likely to go hand-in-hand [37].

We have also shown that the peritoneum from women with endometriosis expressed higher levels of *TGFB1* mRNA transcripts in peritoneum adjacent to endometriosis lesions, when compared to peritoneum distal to endometriosis lesions. This suggests that there is a local disruption of the peritoneum signalling pathways and may indicate that the peritoneum adjacent to endometriosis lesions plays a part in their establishment and survival leading to an integrated microenvironment. We found no difference in expression of *TGFB1* neither between women with and without endometriosis nor between sites of peritoneum in women without endometriosis. However, whether women with endometriosis have an altered TGF-β peritoneal environment that predisposes to endometriosis, or, that the presence of peritoneal endometriosis lesions induce changes in adjacent peritoneum and the peritoneal fluid is not clear. Given that expression of *TGFB1* is significantly different in peritoneum from sites distal to endometriosis lesions compared to sites adjacent to endometriosis lesions but there is no difference in peritoneal expression of *TGFB1* between women with and without endometriosis lesions, it is likely to be the later and not a global change in the peritoneum of women with endometriosis. This increase in *TGFB1* expression may be a result of the increased inflammatory environment induced by the presence of an endometriosis lesion.

Interestingly, we have shown that the peritoneum is responsive to TGF-β signalling but that there was no change in TGF-β receptor expression or Smad 3 expression, suggesting that the sensitivity of the peritoneum to TGF-β remained similar in all comparisons made. Therefore, increasing TGF-β1 levels in women with endometriosis is likely to have significant down stream effects on TGF-β signalling targets within peritoneal tissue.

Lastly, we assessed expression of TGF-β signalling targets in the peritoneum of women with and without endometriosis. We compared peritoneum from women without endometriosis and looked at expression levels between sites of peritoneum prone to developing endometriosis compared to control peritoneum. We found only one gene, *IL10* coding for cytokine interluken-10 (IL-10), to be significantly increased. IL-10 is reported to be increased in the peritoneal fluid and serum of women with endometriosis and increasing IL-10 has been linked to a decrease in CD4+ T lymphocyte activation and therefor decreased immune responses [38], [39]. A mouse model of surgically induced endometriosis demonstrated that IL-10 promoted the growth of endometriosis lesions and this was suggested to be due to IL-10 supressed immunity allowing endometrial implants to develop [38]. However as we found no differences in TGF-β expression or signalling within this tissue type comparison, changes in *IL10* expression may not be TGF-β dependant. Nevertheless an increase in IL-10 expression in peritoneum within the pouch of Douglas may lead to supressed immunity within this local area allowing for ectopic endometrial cells to survive and develop into endometriosis lesions.

When we compared peritoneum from women with endometriosis to women without endometriosis, we saw a significant change in six genes. The significant changes in the expression of TGF-β signalling targets co-insides with the observation that there is significantly higher TGF-β1 within the peritoneal fluid of women with endometriosis. Of those increased, *NOTCH1* is associated with cancer metastasis though TGF-β induced EMT and EMT of endometriotic cells has recently been implicated in the development of endometriosis [40], [41]. This data supports the hypothesis that the peritoneum of women with endometriosis may also be undergoing EMT and this could help facilitate ectopic cell invasion [5]. Transcription factor *CREB1* a key regulator of neurotropic factors such as nerve growth factor (NGF) and brain-derived neurotrophic factor (BDNF) was also significantly increased. NGF and BDNF have recently been described in the context of endometriosis associated pain and regulation of neurogenesis in and around the sites of lesions [28].

*ID1* and *ID3* share a similar pattern of expression and both were significantly increased in the peritoneum of women with endometriosis. *ID1* and *ID3* are essential for angiogenesis and neurogenesis during development and tumor growth, with ID1^+/−^ ID3^−/−^ knockout mice unable to support the growth of tumors [29]. Overexpression of *ID1* has been described in several cancers and has been specifically linked to increases in *VEGFA* gene transcription [42]–[44]. Increased VEGF expression is essential for endometriosis lesion development through the initiation of angiogenesis [45]–[47]. However a role for ID proteins in the aetiology of endometriosis has not been reported.

*MAPK8* that plays a critical role in proliferation, differentiation and apoptosis and MAPK8 activity has been implicated in a range of human diseases including cancer, neurological and inflammatory conditions [30]. Two studies have demonstrated MAPK8 activity in endometriosis and this was linked to increased inflammatory responses [48], [49]. MAPK8 has been shown to inhibit apoptosis though Bad which can also induce glycolysis, a common trait seen in nearly all cancers [50]–[52]. This supports findings from a recent study from our laboratory that demonstrated that there are metabolic changes in the peritoneum of women with endometriosis, consistent with the induction of glycolysis, and which may promote the establishment and progression of endometriosis lesions [53]. CDC6 was also significantly increased. CDC6 is an ATPase associated with cell cycle progression but has not been described in the pathophysiology of endometriosis [31].

Subsequently, we assessed changes in women with endometriosis from sites adjacent to endometriosis compared to sites distal to endometriosis lesions. We found three genes to be significantly increased and one gene to be significantly decreased. NOTCH1 and CREB1 as described above, were significantly increased suggesting that the peritoneum adjacent to endometriosis lesions may be more prone to undergoing TGF-β induced EMT and neurogenesis [27],[40], [41]. Transcription factor *SOX4* was increased in peritoneum adjacent to endometriosis lesions. SOX4 is reported to have impacts on inhibition of apoptosis, increased cell proliferation and EMT, although a role for SOX4 has not been described in the context of endometriosis [32]. Together these results suggests that there are local environmental changes in the peritoneum that surrounds endometriosis lesions that are similar to those seen in tumorigenesis and that these changes may help facilitate endometriosis development and progression.

In this study, we have shown that the peritoneal mesothelium may be responsible for the increased TGF-β levels in women with endometriosis. Furthermore we have shown that expression of several TGF-β signalling targets is altered within the peritoneum of women with endometriosis and these may play a role in lesion development. These data suggest that the peritoneum expresses higher levels of genes associated with tumorigenesis, inflammation and EMT indicating that the peritoneum may contribute to the development of endometriosis by facilitating growth and survival through angiogenesis, cell invasion through EMT and may play a role in endometriosis associated pain though neurogenesis. These data shed new light on how TGF-β signalling plays a significant role in endometriosis and provides new targets that may be beneficial for the management of endometriosis.

## Supporting Information

Data S1 The TGF-β signalling targets array (Quigen, Manchester UK) assays 84 TGF-β regulated genes involved in functional processes, including; differentiation, proliferation, migration, apoptosis and cell cycle control. We made three comparisons using this methodology. Firstly we compared peritoneum from sites prone to developing endometriosis to control sites of peritoneam, in women without endometriosis. Secondly we compared peritoneum from women with endometriosis from sites adjacent and distal to endometriosis lesions. Finally we compared peritoneum from women with endometriosis at sites adjacent to endometriosis lesions to peritoneum from women without disease at sites prone to endometriosis. The overall expression profile for all functionally focused genes assayed is listed here. Genes are grouped according to functionality as listed by the manufacturer in the product specification literature. Due to several genes having a wide functionality, gene responses may be listed in more than one table.
(DOCX)
