# Supplementary material for: The Peritoneum Is Both a Source and Target of TGF-β in Women with Endometriosis
Source: PLoS One. 2014 Sep 10;9(9):e106773. doi: 10.1371/journal.pone.0106773 (PMC4160207; doi:10.1371/journal.pone.0106773)
Supplement: Data S1 — The TGF-β signalling targets array (Quigen, Manchester UK) assays 84 TGF-β regulated genes involved in functional processes, including; differentiation, proliferation, migration, apoptosis and cell cycle control. We made three comparisons using this methodology. Firstly we compared peritoneum from sites prone to developing endometriosis to control sites of peritoneam, in women without endometriosis. Secondly we compared peritoneum from women with endometriosis from sites adjacent and distal to endometriosis lesions. Finally we compared peritoneum from women with endometriosis at sites adjacent to endometriosis lesions to peritoneum from women without disease at sites prone to endometriosis. The overall expression profile for all functionally focused genes assayed is listed here. Genes are grouped according to functionality as listed by the manufacturer in the product specification literature. Due to several genes having a wide functionality, gene responses may be listed in more than one table. (DOCX) [file pone.0106773.s001.docx]

## Supplementary Data

**Data S1:** The TGF-β signalling targets array (Quigen, Manchester UK) assays 84 TGF-β regulated genes involved in functional processes, including; differentiation, proliferation, migration, apoptosis and cell cycle control. We made three comparisons using this methodology. Firstly we compared peritoneum from sites prone to developing endometriosis to control sites of peritoneam, in women without endometriosis. Secondly we compared peritoneum from women with endometriosis from sites adjacent and distal to endometriosis lesions. Finally we compared peritoneum from women with endometriosis at sites adjacent to endometriosis lesions to peritoneum from women without disease at sites prone to endometriosis. The overall expression profile for all functionally focused genes assayed is listed here. Genes are grouped according to functionality as listed by the manufacturer in the product specification literature. Due to several genes having a wide functionality, gene responses may be listed in more than one table.

## TGF-β signalling target expression in women without endometriosis

Comparison of peritoneum from sites prone to developing endometriosis to control sites, in women without endometriosis

| **Differentiation and Development** | | | |
| --- | --- | --- | --- |
| **Gene** | **Symbol** | **Fold change** | **P value** |
| Aryl hydrocarbon receptor interacting protein like 1 | AIPL1 | Undetermined | - |
| Brain-derived neurotrophic factor | BDNF | 1.78 | 0.156 |
| Bromodomain containing 2 | BRD2 | -1.06 | 0.762 |
| CCAAT/enhancer binding protein (C/EBP), beta | CEBPB | -1.04 | 0.726 |
| CAMP responsive element binding protein 1 | CREB1 | -1.16 | 0.139 |
| Beta catenin | CTNNB1 | 1.12 | 0.587 |
| Heat shock protein 40 | DNAJA1 | -1.35 | 0.457 |
| Epithelial membrane protein 1 | EMP1 | -1.04 | 0.677 |
| Endoglin | ENG | -1.44 | 0.435 |
| EPH receptor B2 | EPHB2 | -1.16 | 0.905 |
| Fibronectin 1 | FN1 | 1.13 | 0.783 |
| Hairy and enhancer of split 1 | HES1 | 2.09 | 0.282 |
| Interferon-related developmental regulator 1 | IFRD1 | -1.11 | 0.464 |
| Kruppel-like factor 10 | KLF10 | -1.10 | 0.668 |
| Mitogen-activated protein kinase 14 | MAPK14 | 1.14 | 0.643 |
| Matrix metallopeptidase 2 | MMP2 | 2.46 | 0.221 |
| Myogenic differentiation 1 | MYOD1 | Undetermined | - |
| Nuclear factor of kappa light polypeptide gene enhancer in B-cells inhibitor, alpha | NFKBIA | -1.14 | 0.256 |
| Peroxisome proliferator-activated receptor alpha | PPARA | -1.08 | 0.504 |
| Parathyroid hormone-like hormone | PTHLH | 2.29 | 0.308 |
| PTK2 protein tyrosine kinase 2 | PTK2 | -1.08 | 0.675 |
| Retinoic acid receptor, alpha | RARA | -1.32 | 0.525 |
| Ras homolog gene family, member A | RHOA | 1.06 | 0.865 |
| Runt-related transcription factor 1 | RUNX1 | 1.13 | 0.906 |
| Sonic hedgehog | SHH | Undetermined | - |
| Snail homolog 1 | SNAI1 | 1.06 | 0.934 |
| Transforming growth factor, beta 2 | TGFB2 | 1.02 | 0.927 |
| Transforming growth factor, beta receptor II | TGFBR2 | 1.08 | 0.737 |

Table S1. Fold changes of greater than 1.5 and p-values of less than 0.05 are highlighted in red. Gene expression that was undectable is highlighed in blue. 3 genes involved in differentiation and development were not expressed in this sample set and 4 genes had an increased expression of 50% or greater, although none reached significance.

| **Proliferation and Migration** | | | |
| --- | --- | --- | --- |
| **Gene** | **Symbol** | **Fold change** | **P value** |
| Smooth muscle actin | ACTA2 | 1.13 | 0.959 |
| Activin A receptor I | ACVR1 | 1.17 | 0.247 |
| Activin A receptor II like 1 | ACVRL1 | 1.28 | 0.351 |
| Angiotensinogen | AGT | 2.01 | 0.278 |
| Androgen receptor | AR | 1.42 | 0.390 |
| Activating transcription factor 3 | ATF3 | -1.59 | 0.727 |
| BCL2 like 1 | BCL2L1 | -1.19 | 0.657 |
| Brain-derived neurotrophic factor | BDNF | 1.78 | 0.156 |
| Cell division cycle 6 homolog | CDC6 | 1.35 | 0.739 |
| Cyclin-dependent kinase inhibitor 1B (p27) | CDKN1B | 1.04 | 0.941 |
| Beta catenin | CTNNB1 | 1.12 | 0.587 |
| Epithelial membrane protein 1 | EMP1 | -1.04 | 0.677 |
| Fibronectin 1 | FN1 | 1.13 | 0.783 |
| Furin | FURIN | -1.90 | 0.130 |
| Hairy and enhancer of split 1 | HES1 | 2.09 | 0.282 |
| Heme oxygenase 1 | HMOX1 | -1.06 | 0.405 |
| Interleukin 10 | IL10 | 2.75 | 0.026 |
| Kruppel-like factor 10 | KLF10 | -1.10 | 0.668 |
| Msh homeobox 2 | MSX2 | 1.43 | 0.714 |
| V-myc myelocytomatosis viral oncogene homolog | MYC | -1.34 | 0.454 |
| Nuclear factor of kappa light polypeptide gene enhancer in B-cells inhibitor, alpha | NFKBIA | -1.14 | 0.256 |
| Platelet-derived growth factor alpha polypeptide | PDGFA | -1.10 | 0.864 |
| Plasminogen | PLG | -1.27 | 0.905 |
| Cyclooxygenase 2 | PTGS2 | 3.95 | 0.358 |
| Parathyroid hormone-like hormone | PTHLH | 2.29 | 0.308 |
| PTK2 protein tyrosine kinase 2 | PTK2 | -1.08 | 0.675 |
| PTK2B protein tyrosine kinase 2 beta | PTK2B | -1.69 | 0.261 |
| Plasminogen activator inhibitor type 1 | SERPINE1 | 2.54 | 0.402 |
| Sonic hedgehog | SHH | Undetermined | NA |
| sex determining region Y -box 4 | SOX4 | 2.06 | 0.531 |
| Transforming growth factor, beta 2 | TGFB2 | 1.02 | 0.927 |
| Transforming growth factor, beta receptor II | TGFBR2 | 1.08 | 0.737 |
| Thrombospondin 1 | THBS1 | 1.48 | 0.259 |
| Thioredoxin interacting protein | TXNIP | -1.42 | 0.066 |
| Vascular endothelial growth factor A | VEGFA | 1.46 | 0.374 |

Table S2. Fold changes of greater than 1.5 and p-values of less than 0.05 are highlighted in red. Gene expression that was undectable is highlighed in blue. 1 gene involved in proliferation and migration was not expressed and 9 genes had a change in expression of 50% or greater, although only 1 reached significance.

| **Apoptosis** | | | |
| --- | --- | --- | --- |
| **Gene** | **Symbol** | **Fold change** | **P value** |
| Activin A receptor I | ACVR1 | 1.17 | 0.247 |
| Angiotensinogen | AGT | 2.01 | 0.278 |
| Aryl hydrocarbon receptor interacting protein like 1 | AIPL1 | Undetermined | NA |
| Cyclin-dependent kinase inhibitor 1B (p27) | CDKN1B | 1.04 | 0.941 |
| CCAAT/enhancer binding protein (C/EBP), beta | CEBPB | -1.04 | 0.726 |
| CAMP responsive element binding protein 1 | CREB1 | -1.16 | 0.139 |
| Beta catenin | CTNNB1 | 1.12 | 0.587 |
| Furin | FURIN | -1.90 | 0.130 |
| Growth arrest and DNA-damage-inducible, beta | GADD45B | 1.23 | 0.789 |
| Homocysteine-inducible, endoplasmic reticulum stress-inducible, ubiquitin-like domain member 1 | HERPUD1 | -1.64 | 0.261 |
| Kruppel-like factor 10 | KLF10 | -1.10 | 0.668 |
| Mitogen-activated protein kinase kinase kinase 7 | MAP3K7 | -1.15 | 0.401 |
| Mitogen-activated protein kinase 8 | MAPK8 | -1.04 | 0.130 |
| Msh homeobox 2 | MSX2 | 1.43 | 0.714 |
| Plasminogen | PLG | -1.27 | 0.905 |
| Cyclooxygenase 2 | PTGS2 | 3.95 | 0.358 |
| RAD21 homolog | RAD21 | -1.24 | 0.372 |
| Ras homolog gene family, member A | RHOA | 1.06 | 0.865 |
| Ras homolog gene family, member B | RHOB | -1.89 | 0.251 |
| RING1 and YY1 binding protein | RYBP | 1.45 | 0.279 |
| S100 calcium binding protein A8 | S100A8 | -2.16 | 0.552 |
| Sonic hedgehog | SHH | Undetermined | NA |
| sex determining region Y -box 4 | SOX4 | 2.06 | 0.531 |
| Transforming growth factor, beta 2 | TGFB2 | 1.02 | 0.927 |
| Tumor necrosis factor superfamily, member 10 | TNFSF10 | -1.11 | 0.628 |
| Thioredoxin interacting protein | TXNIP | -1.42 | 0.066 |

Table S3. Fold changes of greater than 1.5 and p-values of less than 0.05 are highlighted in red. Gene expression that was undectable is highlighed in blue. 2 genes involved in apoptosis were not expressed in this sample set and 7 genes had a change in expression of 50% or greater, although none reached significance.

| **Anti-Apoptosis** | | | |
| --- | --- | --- | --- |
| **Gene** | **Symbol** | **Fold change** | **P value** |
| BCL2 like 1 | BCL2L1 | -1.19 | 0.657 |
| Brain-derived neurotrophic factor | BDNF | 1.78 | 0.156 |
| CCAAT/enhancer binding protein (C/EBP), beta | CEBPB | -1.04 | 0.726 |
| Crystallin, alpha B | CRYAB | -1.58 | 0.538 |
| Heme oxygenase 1 | HMOX1 | -1.06 | 0.405 |
| Interleukin 10 | IL10 | 2.75 | 0.026 |
| Nuclear factor of kappa light polypeptide gene enhancer in B-cells inhibitor, alpha | NFKBIA | -1.14 | 0.256 |
| PTK2B protein tyrosine kinase 2 beta | PTK2B | -1.69 | 0.261 |
| Thrombospondin 1 | THBS1 | 1.48 | 0.259 |

Table S4. Fold changes of greater than 1.5 and p-values of less than 0.05 are highlighted in red. 1 gene involved in anti-aoptosis pathways was significnatly increased in this sample set. and 4 genes had a change in expression of 50% or greater, although only 1 reached significance.

| **Cell Cycle** | | | |
| --- | --- | --- | --- |
| **Gene** | **Symbol** | **Fold change** | **P value** |
| Activin A receptor I | ACVR1 | 1.17 | 0.247 |
| Cell division cycle 6 homolog | CDC6 | 1.35 | 0.739 |
| Cyclin-dependent kinase inhibitor 1B (p27) | CDKN1B | 1.04 | 0.941 |
| Growth arrest and DNA-damage-inducible, beta | GADD45B | 1.23 | 0.789 |
| Cyclooxygenase 2 | PTGS2 | 3.95 | 0.358 |
| RAD21 homolog | RAD21 | -1.24 | 0.372 |
| Ras homolog gene family, member B | RHOB | -1.89 | 0.251 |
| Transforming growth factor, beta 2 | TGFB2 | 1.02 | 0.927 |

Table S5. Fold changes of greater than 1.5 and p-values of less than 0.05 are highlighted in red. Gene expression that was undectable is highlighed in blue and 2 genes had a change in expression of 50% or greater, although only 1 reached significance.

## TGF-β signalling target expression in women with endometriosis

Comparison of peritoneum from sites adjacent and distal to endometriosis lesions, from women with endometriosis.

| **Differentiation and Development** | | | |
| --- | --- | --- | --- |
| **Gene** | **Symbol** | **Fold change** | **P value** |
| Aryl hydrocarbon receptor interacting protein like 1 | AIPL1 | Undetermined | NA |
| Brain-derived neurotrophic factor | BDNF | -1.39 | 0.423 |
| Bromodomain containing 2 | BRD2 | -1.74 | 0.594 |
| CCAAT/enhancer binding protein (C/EBP), beta | CEBPB | 1.25 | 0.568 |
| CAMP responsive element binding protein 1 | CREB1 | 1.44 | 0.026 |
| Beta catenin | CTNNB1 | 1.23 | 0.626 |
| Heat shock protein 40 | DNAJA1 | 1.05 | 0.825 |
| Epithelial membrane protein 1 | EMP1 | -1.37 | 0.523 |
| Endoglin | ENG | 1.11 | 0.498 |
| EPH receptor B2 | EPHB2 | -1.72 | 0.211 |
| Fibronectin 1 | FN1 | -1.00 | 0.866 |
| Hairy and enhancer of split 1 | HES1 | 1.23 | 0.690 |
| Interferon-related developmental regulator 1 | IFRD1 | 1.06 | 0.671 |
| Kruppel-like factor 10 | KLF10 | 1.01 | 0.927 |
| Mitogen-activated protein kinase 14 | MAPK14 | 1.42 | 0.052 |
| Matrix metallopeptidase 2 | MMP2 | -1.37 | 0.421 |
| Myogenic differentiation 1 | MYOD1 | Undetermined | NA |
| Nuclear factor of kappa light polypeptide gene enhancer in B-cells inhibitor, alpha | NFKBIA | 1.33 | 0.196 |
| Peroxisome proliferator-activated receptor alpha | PPARA | 1.09 | 0.375 |
| Parathyroid hormone-like hormone | PTHLH | -1.87 | 0.327 |
| PTK2 protein tyrosine kinase 2 | PTK2 | 1.09 | 0.314 |
| Retinoic acid receptor, alpha | RARA | -1.03 | 0.709 |
| Ras homolog gene family, member A | RHOA | -1.10 | 0.533 |
| Runt-related transcription factor 1 | RUNX1 | 1.26 | 0.237 |
| Sonic hedgehog | SHH | Undetermined | NA |
| Snail homolog 1 | SNAI1 | -1.09 | 0.923 |
| Transforming growth factor, beta 2 | TGFB2 | -1.27 | 0.153 |
| Transforming growth factor, beta receptor II | TGFBR2 | -1.06 | 0.812 |

Table S6. Fold changes of greater than 1.5 and p-values of less than 0.05 are highlighted in red. Gene expression that was undectable is highlighed in blue. 3 genes involved in differentiation and development were not expressed and and 3 genes had a change in expression of 50% or greater, although only 1 reached significance in this sample set.

| **Proliferation and Migration** | | | |
| --- | --- | --- | --- |
| **Gene** | **Symbol** | **Fold change** | **P value** |
| Smooth muscle actin | ACTA2 | 1.54 | 0.433 |
| Activin A receptor I | ACVR1 | 1.05 | 0.572 |
| Activin A receptor II like 1 | ACVRL1 | 1.17 | 0.688 |
| Angiotensinogen | AGT | -1.22 | 0.688 |
| Androgen receptor | AR | 1.17 | 0.472 |
| Activating transcription factor 3 | ATF3 | 1.11 | 0.386 |
| BCL2 like 1 | BCL2L1 | 1.37 | 0.101 |
| Brain-derived neurotrophic factor | BDNF | -1.39 | 0.423 |
| Cell division cycle 6 homolog | CDC6 | 1.86 | 0.120 |
| Cyclin-dependent kinase inhibitor 1B (p27) | CDKN1B | 1.27 | 0.558 |
| Beta catenin | CTNNB1 | 1.23 | 0.626 |
| Epithelial membrane protein 1 | EMP1 | -1.37 | 0.523 |
| Fibronectin 1 | FN1 | -1.00 | 0.866 |
| Furin | FURIN | -1.14 | 0.668 |
| Hairy and enhancer of split 1 | HES1 | 1.23 | 0.690 |
| Heme oxygenase 1 | HMOX1 | -1.38 | 0.198 |
| Interleukin 10 | IL10 | 2.16 | 0.364 |
| Kruppel-like factor 10 | KLF10 | 1.01 | 0.927 |
| Msh homeobox 2 | MSX2 | 2.32 | 0.218 |
| V-myc myelocytomatosis viral oncogene homolog | MYC | 1.32 | 0.360 |
| Nuclear factor of kappa light polypeptide gene enhancer in B-cells inhibitor, alpha | NFKBIA | 1.33 | 0.196 |
| Platelet-derived growth factor alpha polypeptide | PDGFA | -1.21 | 0.243 |
| Plasminogen | PLG | 1.29 | 0.135 |
| Cyclooxygenase 2 | PTGS2 | 1.12 | 0.897 |
| Parathyroid hormone-like hormone | PTHLH | -1.87 | 0.327 |
| PTK2 protein tyrosine kinase 2 | PTK2 | 1.09 | 0.314 |
| PTK2B protein tyrosine kinase 2 beta | PTK2B | -1.46 | 0.004 |
| Plasminogen activator inhibitor type 1 | SERPINE1 | 1.35 | 0.607 |
| Sonic hedgehog | SHH | Undetermined | NA |
| sex determining region Y -box 4 | SOX4 | 1.76 | 0.004 |
| Transforming growth factor, beta 2 | TGFB2 | -1.27 | 0.153 |
| Transforming growth factor, beta receptor II | TGFBR2 | -1.06 | 0.812 |
| Thrombospondin 1 | THBS1 | 2.33 | 0.205 |
| Thioredoxin interacting protein | TXNIP | -1.00 | 0.984 |
| Vascular endothelial growth factor A | VEGFA | -1.84 | 0.147 |

Table S7. Fold changes of greater than 1.5 and p-values of less than 0.05 are highlighted in red. Gene expression that was undectable is highlighed in blue. 1 gene involved in proliferation and migration was not expressed and 7 genes had a change in expression of 50% or greater, although only 2 reached significance in this sample set.

| **Apoptosis** | | | |
| --- | --- | --- | --- |
| **Gene** | **Symbol** | **Fold change** | **P value** |
| Activin A receptor I | ACVR1 | 1.05 | 0.572 |
| Angiotensinogen | AGT | -1.22 | 0.688 |
| Aryl hydrocarbon receptor interacting protein like 1 | AIPL1 | Undetermined | NA |
| Cyclin-dependent kinase inhibitor 1B (p27) | CDKN1B | 1.27 | 0.558 |
| CCAAT/enhancer binding protein (C/EBP), beta | CEBPB | 1.25 | 0.568 |
| CAMP responsive element binding protein 1 | CREB1 | 1.44 | 0.026 |
| Beta catenin | CTNNB1 | 1.23 | 0.626 |
| Furin | FURIN | -1.14 | 0.668 |
| Growth arrest and DNA-damage-inducible, beta | GADD45B | 1.17 | 0.534 |
| Homocysteine-inducible, endoplasmic reticulum stress-inducible, ubiquitin-like domain member 1 | HERPUD1 | 1.36 | 0.123 |
| Kruppel-like factor 10 | KLF10 | 1.01 | 0.927 |
| Mitogen-activated protein kinase kinase kinase 7 | MAP3K7 | 1.31 | 0.506 |
| Mitogen-activated protein kinase 8 | MAPK8 | 1.38 | 0.088 |
| Msh homeobox 2 | MSX2 | 2.32 | 0.218 |
| Plasminogen | PLG | 1.29 | 0.135 |
| Cyclooxygenase 2 | PTGS2 | 1.12 | 0.897 |
| RAD21 homolog | RAD21 | 1.45 | 0.128 |
| Ras homolog gene family, member A | RHOA | -1.10 | 0.533 |
| Ras homolog gene family, member B | RHOB | 1.34 | 0.143 |
| RING1 and YY1 binding protein | RYBP | 1.12 | 0.183 |
| S100 calcium binding protein A8 | S100A8 | 1.37 | 0.354 |
| Sonic hedgehog | SHH | Undetermined | NA |
| sex determining region Y -box 4 | SOX4 | 1.76 | 0.004 |
| Transforming growth factor, beta 2 | TGFB2 | -1.27 | 0.153 |
| Tumor necrosis factor superfamily, member 10 | TNFSF10 | 1.02 | 0.805 |
| Thioredoxin interacting protein | TXNIP | -1.00 | 0.984 |

Table S8. Fold changes of greater than 1.5 and p-values of less than 0.05 are highlighted in red. Gene expression that was undectable is highlighed in blue. 2 genes involved in apoptosis were not expressed and 2 genes had a change in expression of 50% or greater, although only 1 reached significance.

| **Anti-Apoptosis** | | | |
| --- | --- | --- | --- |
| **Gene** | **Symbol** | **Fold change** | **P value** |
| BCL2 like 1 | BCL2L1 | 1.37 | 0.101 |
| Brain-derived neurotrophic factor | BDNF | -1.39 | 0.423 |
| CCAAT/enhancer binding protein (C/EBP), beta | CEBPB | 1.25 | 0.568 |
| Crystallin, alpha B | CRYAB | -1.05 | 0.663 |
| Heme oxygenase 1 | HMOX1 | -1.38 | 0.198 |
| Interleukin 10 | IL10 | 2.16 | 0.364 |
| Nuclear factor of kappa light polypeptide gene enhancer in B-cells inhibitor, alpha | NFKBIA | 1.33 | 0.196 |
| PTK2B protein tyrosine kinase 2 beta | PTK2B | -1.46 | 0.004 |
| Thrombospondin 1 | THBS1 | 2.33 | 0.205 |

Table S9. Fold changes of greater than 1.5 and p-values of less than 0.05 are highlighted in red. 1 gene involved in anti-aoptosis pathways was significnatly increased and 2 genes had a change in expression of 50% or greater, although they did not reached significance in this sample set .

| **Cell Cycle** | | | |
| --- | --- | --- | --- |
| **Gene** | **Symbol** | **Fold change** | **P value** |
| Activin A receptor I | ACVR1 | 1.05 | 0.572 |
| Cell division cycle 6 homolog | CDC6 | 1.86 | 0.120 |
| Cyclin-dependent kinase inhibitor 1B (p27) | CDKN1B | 1.27 | 0.558 |
| Growth arrest and DNA-damage-inducible, beta | GADD45B | 1.17 | 0.534 |
| Cyclooxygenase 2 | PTGS2 | 1.12 | 0.897 |
| RAD21 homolog | RAD21 | 1.45 | 0.128 |
| Ras homolog gene family, member B | RHOB | 1.34 | 0.143 |
| Transforming growth factor, beta 2 | TGFB2 | -1.27 | 0.153 |

Table S10. Fold changes of greater than 1.5 and p-values of less than 0.05 are highlighted in red. 1 gene had a change in expression of 50% or greater, although it did not reached significance.

## TGF-β signalling targets expression in women with endometriosis compared to women without

Comparison of peritoneum from women with endometriosis at sites adjacent to endometriosis lesions to peritoneum from women without disease at sites prone to endometriosis.

| **Differentiation and Development** | | | |
| --- | --- | --- | --- |
| **Gene** | **Symbol** | **Fold change** | **P value** |
| Aryl hydrocarbon receptor interacting protein like 1 | AIPL1 | Undetermined | NA |
| Brain-derived neurotrophic factor | BDNF | -2.64 | 0.219 |
| Bromodomain containing 2 | BRD2 | -1.67 | 0.613 |
| CCAAT/enhancer binding protein (C/EBP), beta | CEBPB | 1.42 | 0.253 |
| CAMP responsive element binding protein 1 | CREB1 | 1.27 | 0.009 |
| Beta catenin | CTNNB1 | 1.78 | 0.416 |
| Heat shock protein 40 | DNAJA1 | 1.15 | 0.635 |
| Epithelial membrane protein 1 | EMP1 | 1.03 | 0.840 |
| Endoglin | ENG | 1.34 | 0.496 |
| EPH receptor B2 | EPHB2 | -1.12 | 0.576 |
| Fibronectin 1 | FN1 | 1.09 | 0.993 |
| Hairy and enhancer of split 1 | HES1 | 1.01 | 0.964 |
| Interferon-related developmental regulator 1 | IFRD1 | 1.29 | 0.165 |
| Kruppel-like factor 10 | KLF10 | 1.40 | 0.164 |
| Mitogen-activated protein kinase 14 | MAPK14 | 1.06 | 0.700 |
| Matrix metallopeptidase 2 | MMP2 | -1.35 | 0.480 |
| Myogenic differentiation 1 | MYOD1 | Undetermined | NA |
| Nuclear factor of kappa light polypeptide gene enhancer in B-cells inhibitor, alpha | NFKBIA | 1.10 | 0.875 |
| Peroxisome proliferator-activated receptor alpha | PPARA | 1.16 | 0.543 |
| Parathyroid hormone-like hormone | PTHLH | -1.35 | 0.334 |
| PTK2 protein tyrosine kinase 2 | PTK2 | 1.10 | 0.576 |
| Retinoic acid receptor, alpha | RARA | 1.32 | 0.187 |
| Ras homolog gene family, member A | RHOA | -1.43 | 0.172 |
| Runt-related transcription factor 1 | RUNX1 | 1.31 | 0.328 |
| Sonic hedgehog | SHH | Undetermined | NA |
| Snail homolog 1 | SNAI1 | 1.28 | 0.387 |
| Transforming growth factor, beta 2 | TGFB2 | -1.25 | 0.389 |
| Transforming growth factor, beta receptor II | TGFBR2 | 1.13 | 0.700 |

Table S11. Fold changes of greater than 1.5 and p-values of less than 0.05 are highlighted in red. Gene expression that was undectable is highlighed in blue. 3 genes involved in differentiation and development were not expressed and 3 genes had a change in expression of 50% or greater but did not reach significnane, and 1 gene was significantly increased in this sample set.

| **Proliferation and Migration** | | | |
| --- | --- | --- | --- |
| **Gene** | **Symbol** | **Fold change** | **P value** |
| Smooth muscle actin | ACTA2 | -1.96 | 0.219 |
| Activin A receptor I | ACVR1 | 1.10 | 0.576 |
| Activin A receptor II like 1 | ACVRL1 | -1.07 | 0.780 |
| Angiotensinogen | AGT | -1.95 | 0.416 |
| Androgen receptor | AR | 1.02 | 0.857 |
| Activating transcription factor 3 | ATF3 | 1.86 | 0.608 |
| BCL2 like 1 | BCL2L1 | 1.35 | 0.330 |
| Brain-derived neurotrophic factor | BDNF | -2.64 | 0.219 |
| Cell division cycle 6 homolog | CDC6 | 3.18 | 0.046 |
| Cyclin-dependent kinase inhibitor 1B (p27) | CDKN1B | 1.11 | 0.497 |
| Beta catenin | CTNNB1 | 1.78 | 0.416 |
| Epithelial membrane protein 1 | EMP1 | 1.03 | 0.840 |
| Fibronectin 1 | FN1 | 1.09 | 0.993 |
| Furin | FURIN | 1.35 | 0.483 |
| Hairy and enhancer of split 1 | HES1 | 1.01 | 0.964 |
| Heme oxygenase 1 | HMOX1 | 1.23 | 0.791 |
| Interleukin 10 | IL10 | 1.20 | 0.453 |
| Kruppel-like factor 10 | KLF10 | 1.40 | 0.164 |
| Msh homeobox 2 | MSX2 | 4.41 | 0.096 |
| V-myc myelocytomatosis viral oncogene homolog | MYC | 1.68 | 0.206 |
| Nuclear factor of kappa light polypeptide gene enhancer in B-cells inhibitor, alpha | NFKBIA | 1.10 | 0.875 |
| Platelet-derived growth factor alpha polypeptide | PDGFA | -1.17 | 0.407 |
| Plasminogen | PLG | 1.93 | 0.319 |
| Cyclooxygenase 2 | PTGS2 | 3.68 | 0.259 |
| Parathyroid hormone-like hormone | PTHLH | -1.35 | 0.334 |
| PTK2 protein tyrosine kinase 2 | PTK2 | 1.10 | 0.576 |
| PTK2B protein tyrosine kinase 2 beta | PTK2B | 1.01 | 0.962 |
| Plasminogen activator inhibitor type 1 | SERPINE1 | 1.32 | 0.714 |
| Sonic hedgehog | SHH | Undetermined | NA |
| sex determining region Y -box 4 | SOX4 | -1.35 | 0.485 |
| Transforming growth factor, beta 2 | TGFB2 | -1.25 | 0.389 |
| Transforming growth factor, beta receptor II | TGFBR2 | 1.13 | 0.700 |
| Thrombospondin 1 | THBS1 | 1.35 | 0.473 |
| Thioredoxin interacting protein | TXNIP | 1.37 | 0.054 |
| Vascular endothelial growth factor A | VEGFA | -2.18 | 0.149 |

Table S12. Fold changes of greater than 1.5 and p-values of less than 0.05 are highlighted in red. Gene expression that was undectable is highlighed in blue. 1 gene involved in proliferation and migration was not expressed and 10 genes had a change in expression of 50% or greater but only 1 gene reach significnane in this sample set.

| **Apoptosis** | | | |
| --- | --- | --- | --- |
| **Gene** | **Symbol** | **Fold change** | **P value** |
| Activin A receptor I | ACVR1 | 1.10 | 0.576 |
| Angiotensinogen | AGT | -1.95 | 0.416 |
| Aryl hydrocarbon receptor interacting protein like 1 | AIPL1 | Undetermined | NA |
| Cyclin-dependent kinase inhibitor 1B (p27) | CDKN1B | 1.11 | 0.497 |
| CCAAT/enhancer binding protein (C/EBP), beta | CEBPB | 1.42 | 0.253 |
| CAMP responsive element binding protein 1 | CREB1 | 1.27 | 0.009 |
| Beta catenin | CTNNB1 | 1.78 | 0.416 |
| Furin | FURIN | 1.35 | 0.483 |
| Growth arrest and DNA-damage-inducible, beta | GADD45B | 1.13 | 0.632 |
| Homocysteine-inducible, endoplasmic reticulum stress-inducible, ubiquitin-like domain member 1 | HERPUD1 | 1.54 | 0.104 |
| Kruppel-like factor 10 | KLF10 | 1.40 | 0.164 |
| Mitogen-activated protein kinase kinase kinase 7 | MAP3K7 | 1.12 | 0.795 |
| Mitogen-activated protein kinase 8 | MAPK8 | 1.31 | 0.037 |
| Msh homeobox 2 | MSX2 | 4.41 | 0.096 |
| Plasminogen | PLG | 1.93 | 0.319 |
| Cyclooxygenase 2 | PTGS2 | 3.68 | 0.259 |
| RAD21 homolog | RAD21 | 1.38 | 0.188 |
| Ras homolog gene family, member A | RHOA | -1.43 | 0.172 |
| Ras homolog gene family, member B | RHOB | 1.94 | 0.068 |
| RING1 and YY1 binding protein | RYBP | 1.08 | 0.748 |
| S100 calcium binding protein A8 | S100A8 | 2.25 | 0.179 |
| Sonic hedgehog | SHH | Undetermined | NA |
| sex determining region Y -box 4 | SOX4 | -1.35 | 0.485 |
| Transforming growth factor, beta 2 | TGFB2 | -1.25 | 0.389 |
| Tumor necrosis factor superfamily, member 10 | TNFSF10 | 1.25 | 0.135 |
| Thioredoxin interacting protein | TXNIP | 1.37 | 0.054 |

Table S13. Fold changes of greater than 1.5 and p-values of less than 0.05 are highlighted in red. Gene expression that was undectable is highlighed in blue. 2 genes involved in apoptosis were not expressed and 7 genes had a change in expression of 50% or greater but did not reach significnane, and 2 gene was significantly increased in this sample set.

| **Anti-Apoptosis** | | | |
| --- | --- | --- | --- |
| **Gene** | **Symbol** | **Fold change** | **P value** |
| BCL2 like 1 | BCL2L1 | 1.35 | 0.330 |
| Brain-derived neurotrophic factor | BDNF | -2.64 | 0.219 |
| CCAAT/enhancer binding protein (C/EBP), beta | CEBPB | 1.42 | 0.253 |
| Crystallin, alpha B | CRYAB | -1.12 | 0.592 |
| Heme oxygenase 1 | HMOX1 | 1.23 | 0.791 |
| Interleukin 10 | IL10 | 1.20 | 0.453 |
| Nuclear factor of kappa light polypeptide gene enhancer in B-cells inhibitor, alpha | NFKBIA | 1.10 | 0.875 |
| PTK2B protein tyrosine kinase 2 beta | PTK2B | 1.01 | 0.962 |
| Thrombospondin 1 | THBS1 | 1.35 | 0.473 |

Table S14. Fold changes of greater than 1.5 and p-values of less than 0.05 are highlighted in red. 1 genes had a change in expression of 50% or greater but did not reach significnane this sample set.

| **Cell Cycle** | | | |
| --- | --- | --- | --- |
| **Gene** | **Symbol** | **Fold change** | **P value** |
| Activin A receptor I | ACVR1 | 1.10 | 0.576 |
| Cell division cycle 6 homolog | CDC6 | 3.18 | 0.046 |
| Cyclin-dependent kinase inhibitor 1B (p27) | CDKN1B | 1.11 | 0.497 |
| Growth arrest and DNA-damage-inducible, beta | GADD45B | 1.13 | 0.632 |
| Cyclooxygenase 2 | PTGS2 | 3.68 | 0.259 |
| RAD21 homolog | RAD21 | 1.38 | 0.188 |
| Ras homolog gene family, member B | RHOB | 1.94 | 0.068 |
| Transforming growth factor, beta 2 | TGFB2 | -1.25 | 0.389 |

Table S15. Fold changes of greater than 1.5 and p-values of less than 0.05 are highlighted in red. 3 genes had a change in expression of 50% or greater but only 1 gene reach significnane in this sample set.
